# An Evidence Accumulation Account of Masked Translation Priming in Two Bilingual Populations

**DOI:** 10.3390/brainsci13071066

**Published:** 2023-07-13

**Authors:** Camille Scrimshire, Sara Alicia Amador, Andrea González-García Aldariz, Galilea Meza, Pablo Gomez

**Affiliations:** 1 Psychology Department, California State University, San Bernardino-Palm Desert Campus, 37500 Cook St., Palm Desert, CA 92211, USA; 007730925@coyote.csusb.edu (C.S.); 007598377@coyote.csusb.edu (S.A.A.);; 2 Centro de Investigación en Ciencia Cognitiva, Universidad Nebrija, 28240 Madrid, Spain; agonzalezga@nebrija.es; 3 Psychology Department, Skidmore College, Saratoga Springs, NY 12866, USA

**Keywords:** bilingualism, lexical access, masked priming, translation priming

## Abstract

This manuscript addresses the phenomenon of masked priming and the cognitive process of switching from Spanish to English while reading in sequential bilinguals compared to heritage speakers. A lexical decision task was employed in the present study with masked translation priming, which serves as a valuable tool for elucidating the orthographic and lexical processes involved in the initial stages of reading. This study builds upon previous research conducted on monolingual masked priming, which consistently demonstrates shifts in the response time (RT) distributions when comparing related and unrelated primes. Within the framework of a diffusion model, we implemented two theoretical positions. First, we posited that translation priming operates at the orthographic level, resulting in enhanced efficiency during the encoding process. Second, we explored the possibility that translation priming operates at the semantic level, influencing the accumulation of evidence during the lexical decision task. The findings of the present study indicate that translation priming elicits outcomes similar to those observed in monolingual priming paradigms. Specifically, we observed that translation priming facilitation is manifested as shifts in the RT distributions. These findings are interpreted to suggest that the benefits derived from the encoding process are not specific to the accessed lexicon following a brief stimulus presentation.

## 1. Introduction

A primary concern of bilingualism research has been to describe the interaction between the two lexicons available to bilingual individuals. Hence, researchers have enthusiastically argued about how one language might facilitate or inhibit the processing of the other language during the early moments of reading. Adding to this literature, the present work aims to use state-of-the-art methodology to explore the nature of lexical access in bilingual individuals. We do this by examining performance in a lexical decision task with masked priming by two rather distinct types of bilinguals: sequential and heritage speakers.

**Figure 1:**
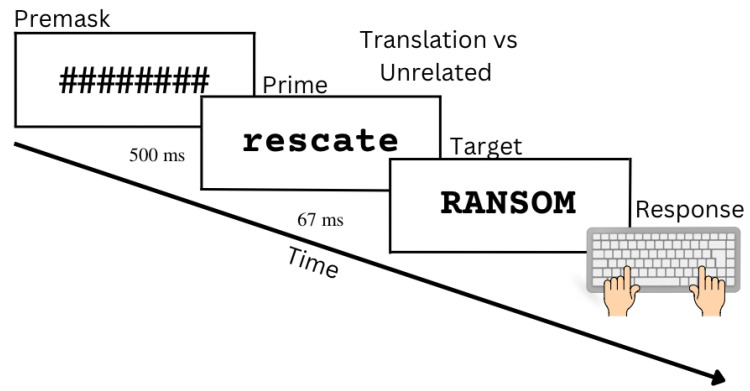
Representation of the timing of the trials.

*Lexical access* can be understood, using the seminal Balota and Chumbley [1] description, as mapping perceptual features to an internal representation of words. A common experimental procedure to explore lexical access is the lexical decision task and its extension, the masked priming procedure. Lexical decisions involve the presentation of strings of letters that can form words (e.g., DOCTOR) or non-words (e.g., DOTHIR). A particularly fruitful paradigm within lexical decisions is a technique originally developed by Forster and Davis [2] called masked priming (see [3,4], for reviews). Masked priming allows researchers to investigate orthographic, semantic, identity, and phonological effects in visual word recognition [3,4,5,6]. This technique typically includes a mask of characters (######) that appears for about 500 ms followed by a prime that only lasts for 67 ms or less, which in turn is followed by the target string of letters that participants make a lexical decision about (see Figure 1). The prime is a stimulus that could influence the response to the target [2]; therefore, researchers manipulate the relationship between primes and targets to explore mechanisms of interest. In this case, the focus will be on translation priming.

Translation priming refers to a setup in which the prime word, which is presented in one language, is followed by the presentation of a target word in another language [7]. Due to the etymological roots of words in the languages of interest (in our case Spanish and English), there can be different degrees of orthographic overlap between the prime and the target (see [8] for an examination of the different types of primes); examples can be seen in Table 1. This research had four types of prime–TARGET relationships: (1) identity cognate primes that share. meaning and orthography with target words (e.g., doctor-DOCTOR) (the term *identity* instead of *identical* is used to convey that the letter case of the prime and target is different, so strictly speaking, the two stimuli are not identical); (2) non-identity cognate primes that share meaning, and a similar orthography with the target (e.g., sal-SALT); (3) non-cognate translation primes that do not share orthography but do share meaning (e.g., silla-CHAIR); and (4) unrelated primes that do not share orthography or meaning with the target (e.g., casa-PLAY).

Translation priming consistently yields faster response times to the targets relative to unrelated primes, particularly for L1 primes and L2 targets [9,10,11] and for proficient bilinguals in both directions [12]. This is the case for identity (e.g., doctor-DOCTOR) and non-identity cognates (e.g., plato-PLATE), which share orthographic and semantic representation [13]. More intriguingly, even words that are not orthographically related, but are still translation pairs, such as caballo and HORSE, are thought to activate shared semantic representations because of their conceptual relationship [8], as is evident by the facilitation of responses in these cases as well.

### 1.1. Masked Priming as Savings

To explain masked translation priming, it is useful to consider the models of masked priming in the monolingual literature. An influential account is that masked identity prime words have an encoding advantage over unrelated primes because of how they activate letter representation at an abstract level. This explanation, termed the *savings account* [3], was implemented within a perceptual accumulation framework by Gomez et al. [14] and Gomez and Perea [6]. The main insight is that the activation of letter representation for the target occurs upon exposure to the prime, providing savings in the letter encoding process [3,5].

The savings hypothesis has not been a focus with translation masking priming; however, some adaptation of the idea is in considering that the facilitation from cognate primes is higher than from non-cognate primes. This evidence suggests that the savings account would apply to translation priming. This facilitation has been noted as evidence of the non-selective nature of bilinguals when accessing the lexicon [8].

### 1.2. Cross-Language Inhibitory Effects

While the facilitation effects of masked translation priming are quite robust and not in question, it must be noted that switching from one language to another comes at a processing cost. This observation has yielded a vibrant literature exploring the generality vs. specificity of cognitive control for bilinguals [15,16,17]. Perhaps the dominant view, until recently, has been that the reason for inhibition in code switching is external to the mechanisms that are specific to language, and that the cost is due to executive control factors, such as those related to decision making in laboratory tasks [15,17,18,19].

The inhibition that takes place in language switching comprehension for bilinguals has been found to be asymmetrical in that the cost for the switch from L1 to L2 is greater, while in language switching production, the cost is greater switching from L2 to L1 [16,18]. In the present work, the goal is to explore if evidence of inhibition can be uncovered within translation-masked priming. To do so, we use distributional analyses interpreted under the framework of evidence accumulation models. Hence, an explanation of such models and their relationship with masked priming is in order.

### 1.3. Perceptual Accumulation Models

The task used in this study is the lexical decision task, which, in its canonical form, involves two choices: the response “word” and the response “nonword”. Two-choice tasks have a long tradition in cognitive psychology, and one of the most fruitful ways to understand performance in these tasks is to conceptualize it as an evidence accumulation process. One of the main advantages of these conceptualizations is their ability to account for speed and accuracy simultaneously, as well as to allow us to make inferences about processes based on RT distributions. Importantly, RT distributions provide stronger constraints for hypotheses than mean RTs.

In the last two decades, perceptual accumulation models have been useful tools to understand performance in two-choice tasks. Although some researchers (e.g., [17]) have used this type of model to explore issues related to bilingualism, the bulk of the work has been in a monolingual context.

Of particular relevance to this research is the work conducted with the drift-diffusion model [20] to account for word/non-word decisions (lexical decision task [21,22]). The drift-diffusion model (DDM from now on) assumes that tokens of evidence are accumulated over time towards either the “word” or the “non-word” decision boundaries. This evidence accumulation is stochastic, meaning that it does not always go in the same direction, and once it hits the decision boundary, a motor response is initiated (e.g., pressing the button for the “word” response).

For the purposes of this research, there are two parameters of the model that are most relevant: 1. The drift rate parameter, which is the rate at which evidence is accumulated. Ratcliff et al. 2004 showed that variables such as word frequency can be accounted for by this parameter. Unmasked priming also affects this parameter. 2. The $T_{er}$ parameter, which represents the encoding time (i.e., the time taken to transform the sensory information into lexical information) and response execution (the “e” in $T_{er}$ stands for *encoding* and the “r” in $T_{er}$ stands for *response execution*).

A major advantage of the model is that these two parameters account for quite different patterns of data. Differences in the drift rate yield effects in the accuracy rate, and also change the shape of the RT distribution. On the other hand, changes in the $T_{er}$ parameter produce a shift in the distribution with negligible changes in accuracy. Importantly for this research, the model-based analyses indicate an encoding advantage of identity primes [5,6,14]. This is captured by the $T_{er}$ parameter and yields equal effects across all quantiles of the RT distribution.

While the diffusion model is not a model of lexical access, and certainly not a model of bilingualism, it could be useful (“All models are wrong, but some are useful.—George Box”) to implement two theoretical positions about masked priming. These two plausible competing implementations within the drift-diffusion model were proposed by Gomez et al. [14]. In one implementation of the model, the $T_{er}$ parameter is thought to be affected by the priming effect. In the competing implementation of the model, the drift rate parameter is thought to be affected by the priming effect [6,23]. These implementations within the model assume that RTs are a result of the sum of the three processes: encoding, evidence accumulation, and response execution. The encoding and response execution are captured by the $T_{er}$ parameter, and the evidence accumulation is described principally by the drift rate, which is the average rate of evidence accumulation. The difference in RTs from identity and unrelated masked primes have been accounted for by a change in the $T_{er}$ parameter (encoding time and response execution), while there are no differences in the drift rate parameter [5,14]. Ong et al. [17] used the diffusion model to explain the drift rate and non-decision time in their study and found both parameters to be impacted, which revealed a hybrid interpretation in that the cost is due to both language switching and interference in task switching from previous trials. The task switch cost is consistent with interference from trial-to-trial task switching [19]. Language switch costs when considering the RT distribution show an effect on both the drift rate and $T_{er}$ parameters [17].

In a monolingual setting, the differences in RTs between the identity and unrelated prime conditions reflect a shift rather than a change in shape in the distributions. This has been interpreted as support for the savings account hypothesis [3,6,14,24]. Briefly stated, the savings account refers to a head start in the orthographic processes due to the prime. It is important to note that this account does not pertain to a head start in the evidence accumulation process. Furthermore, it is crucial to acknowledge that the data obtained. in this type of experiment arises from the convolution of multiple processes, including strategic, motor, orthographic, and semantic processes. Priming effects observed in the study reflect the overall facilitation or inhibition that emerges from the interaction between various types of primes and targets. Therefore, describing these phenomena solely as “encoding” is a simplification imposed by the data and the model. Furthermore, it is important to recognize that this encoding process is specific to the task at hand, which in this study, is the lexical decision task. In a monolingual setting, there is evidence from De Wit and Kinoshita [25] indicating that masked semantic priming effects are task dependent. Therefore, it is crucial to consider the influence of the task when interpreting and generalizing the findings of this study.

The main question in this work is whether translation priming yields similar effects (a shift in the RT distribution) as monolingual identity priming. To address this question, we explore the masked translation priming effects using cognate and non-cognate translations as prime–TARGET pairs.

### 1.4. Overview of the Experiments

Bilingualism is a complex phenomenon, and a taxonomy of bilingualism is not simple because factors such as fluency, timing of acquisition, and others come into play. In bilingualism research, it is common to say that “no two bilinguals are the same” (e.g., [26]). In the present study, we compare two different types of bilinguals: sequential bilinguals and heritage speakers.

In Experiment 1, we used participants who can be described as sequential bilinguals [27] that speak English as a second language. Their first language is Spanish, and they live in Spanish-speaking countries. These participants fluently read and write in their first language. We collected the data from this group through the online system prolific.com.

In Experiment 2, on the other hand, we used participants who can be described as heritage speakers of Spanish. A heritage speaker is defined as someone who learned a language from birth that is not the dominant language in the country where they live; hence, they are considered a linguistic minority [28]. In our study, the participants in Experiment 2 are Spanish heritage speakers who learned Spanish at home but live in Southern California, where English is the dominant language, and they attended university where English is the only language of instruction. As is the case with many heritage speakers, their fluency in Spanish varies, and some might not be able to speak it fluently, but we ensured that they can understand it (see Methods Section).

We found the comparison of translation priming effects in these two groups of theoretical interest because the sequential bilingual subjects have well-formed lexical representations and orthographic processing of Spanish words in a way that heritage speakers do not.

## 2. Experiment 1

### 2.1. Methods

#### 2.1.1. Participants

Both Expreriments were performed in accordance with the Declaration of Helsinki and the APA code of conduct, and approved by the Institutional Review Board at California State University San Bernardino (IRB approval: IRB-FY2023-10 *Interaction of Sensory and Response Processes in Decision Making*).

Sixty-two participants were recruited (30 self-reported females, 32 self-reported males) with a mean age of 27.0 years (SD = 9.7, age range = 19–73 years) via the online platform Prolific Academia (https://prolific.co, accessed on 8 June 2023 ). Of note, the 73-year-old participant was an outlier in terms of age, but her performance was similar to the rest of the sample.

With this sample size, we obtained 13,268 observations in the critical comparison (translation vs. unrelated primes), which is in line with Brysbaert and Stevens [29] for small-sized effects. We used Prolific Academia’s filtering functions with the following criteria: a gender-balanced panel, participants whose first language is Spanish, participants who report both their country of origin and their country of residence as a Spanish-speaking country (we defined Spanish-speaking as Spain and all countries in Latin America except for Brazil), and self-reported proficiency in English. Note that we filtered for participants with more than 75% accuracy in the lexical decision task, which was performed in English. This filtering process removed four participants from the analyses.

#### 2.1.2. Materials

##### Target Items

Angele et al. [5] demonstrated that the online studies on the Prolific platform can yield high-quality masked priming data. The targets for the lexical decision tasks were the same as those used by Angele et al. [5], which were as follows: 215 six-letter English words from the English Lexicon Project [30], with mean Zip frequency [31] of 3.8 (range 1.9–5.5), and mean OLD20 [32] of 2.1 (range 1.4–3). We also selected 239 matched, orthographically legal (in English) six-letter non-words.

The reason for the slight discrepancy between the number of words and non-words was that we wanted to utilize as many of the Angele et al. [5]’s items as possible, which consisted of 240 words and 240 non-words. However, one non-word was eliminated as it created a Spanish word and 25 words were removed because we could not find a single-word equivalent translation (e.g., to convey the English word “fumble” in Spanish, one would say “dejar caer”).

##### Primes

For each word target, we created a translation prime (e.g., hilo for the target THREAD) and an unrelated prime (e.g., viajar); the unrelated prime was a translation prime for another word from the list. For the non-word targets, we also used words in Spanish from the list as primes. All counterbalanced lists can be found in the online repository (https://osf.io/str9c/, accessed on 29 June 2023).

#### 2.1.3. Procedure

We performed a masked priming procedure with the following structure: a pre-mask (########) was presented for 500 ms, then a prime (a word in Spanish) was presented for 67 ms, which was followed by the presentation of a TARGET string of letters (see Figure 1). After the presentation of the TARGET, participants then decided if the string of letters represented an English word by pressing **z** for non-word and **m** for word for their response. The trial timed out after 2 s, and such trials were not used for the analyses presented below. The experiment took about 15 min.

Upon completing the lexical decision task, participants were debriefed and thanked.

#### 2.1.4. Data Analysis

The data inclusion/exclusion plan was as follows: we pre-planned for 60 participants with the features described above. Given that the participants reported Spanish to be their first language and that they were located in Spanish-speaking countries, no Spanish test was administered. The criterion for using the data was 75% correct overall performance, which was achieved by all but four participants. Since the trial timed out at 2 s, we decided not to set cutoffs for the RTs.

The data analysis plan was as follows:

We first examined whether there was evidence for a masked priming effect on the RT and, if so, in which direction. We used Bayes Factors as our tool for statistical inference; then, we generated delta plots for the latency data. To conclude, we interpreted these results within the evidence accumulation framework.

### 2.2. Results

Table 2 shows the mean RT and the mean accuracy for all types of stimuli. As can be seen, the high accuracy across all the conditions indicates that participants were able to carry out the task accurately and with RTs in line with other published data. Numerically, the priming effects were evident; and the sizes of the priming effects reflect the level of similarity between the primes and targets: 61 ms for identity primes, 35 ms for cognates, and 9 ms for non-cognates (all faster compared to unrelated controls).

The inferential method employed in this study was Bayes Factors, which provide a quantitative measure of the evidence supporting one model over another [33]. Specifically, our aim was to assess whether there is empirical support for a priming effect on response time (RT). For this purpose, three models were considered: (i) a null model ($M_{0}$) assuming the absence of a priming effect, included for completeness; (ii) a main effect only model ($M_{m}$), assuming a priming effect but no interaction with the type of translation prime (cognate vs. non-cognate); and (iii) a full model ($M_{1}$) that includes an interaction term between the type of translation and priming effects.

Based on the computed Bayes Factors (BFs) in this study, we can conclude that the null model, positing the absence of a priming effect, can be rejected. The BF for the main effect model relative to the null model was $5\times 10^{17}$, indicating substantially stronger evidence in favor of the former. However, the comparison between the full model and the main effect model was more relevant to the research question at hand. The BF for the full model relative to the main effect model was 65, indicating that the full model was 65 times more supported by the data than the main effects model.

### 2.3. Distributional Analyses

In cognitive psychology, there is a long tradition of analyzing latency data with methods that go beyond the mean RT. In order to investigate the priming effects associated with translation priming, we conducted a comparative analysis of the empirical reaction time (RT) distributions. Specifically, we utilized delta plots to characterize the differences in latency distributions. It is important to note that this method lacks established inferential properties; thus, our subsequent discussion focuses exclusively on qualitative effects.

Delta plots are a commonly utilized tool for visualizing the temporal evolution of a latency effect, such as identity priming or task effects [34,35]. The construction of delta plots involves the following steps:

The first step in constructing delta plots involves obtaining the reaction times (RTs) for correct responses at specified quantiles (e.g., 0.1, 0.3, 0.5, 0.7, and 0.9) for each participant in the conditions being compared. In the second step, the RTs obtained in the first step were averaged across participants, which are also referred to as vincentiles in the RT literature [36]. In the third step, the average RT between the two conditions was computed for each quantile in the vincentiles. The differences between these averages, the delta, were then calculated while preserving the sign. Finally, in the fourth step, a plot was created where each quantile was represented by a point, with the averages plotted on the *x*-axis and the delta on the *y*-axis.

It is important to note that delta plots, as described above, are based on residual quantiles (i.e., the RTs at quantiles in condition A minus condition B). This makes them useful for providing insight into the temporal dynamics of an effect, particularly when viewed through the lens of process models. For instance, a flat line at y = 50 ms would indicate a 50 ms shift in the RT distributions, which may suggest faster encoding times in evidence accumulation models (see [14] for an example of such an interpretation in the standard masked priming technique). On the other hand, an ascending function would suggest that the effect grows for slower responses, which could be interpreted as a difference in the rate of evidence accumulation.

As previously noted, delta plots require the comparison of two conditions. In our examination translation priming, it is logical to conduct distinct comparisons for each type of translation, which are presented in separate lines. Specifically, we focus on the difference between the translation primes and their controls for cognate and non-cognate translations. Importantly, given the small number of identity translation primes (doctor-DOCTOR) in the materials, we collapse the identity and non-identity cognates into the “cognate” category.

**Figure 2:**
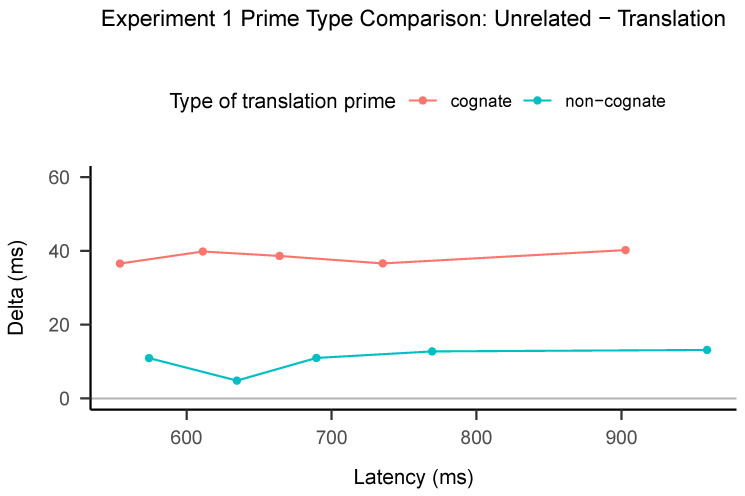
Delta plot for Experiment 1.

As can be seen in Figure 2, the delta plots for both cognate and non-cognate translation primes are straight horizontal lines. Recall that these plots are constructed by calculating the difference between the translation primes and their controls at the 0.1, 0.3, 0.5, 0.7, and 0.9 quantiles; hence, the graph shows two lines.

Most importantly, the delta plot from this experiment has the same main feature as delta plots found by Gomez and Perea [6] and Angele et al. [5]: a flat line. This is consistent with the assumption that the encoding process benefits from a head start in the encoding process.

## 3. Experiment 2

Would the masked priming effect be qualitatively similar in sequential bilinguals as in heritage speakers that have little to no exposure to written Spanish (the language of the prime)? To explore this question, we recruited heritage speakers of Spanish from Southern California (particularly, the Coachella Valley).

### 3.1. Method

The materials and trial structure were identical to Experiment 1.

#### 3.1.1. Participants

Twenty-two participants from the California State University, Palm Desert Campus community took part in this Experiment. We recruited via flyers and word of mouth for bilingual students. The Palm Desert Campus serves a large number of first-generation Americans and DACA recipients; hence, there is a large proportion of Spanish–English bilingual students.

To include the participants in the analyses, we established five criteria: (1) In the language history survey, they must indicate that either Spanish was their first language, or they were simultaneously exposed to English and Spanish. (2) In the language history survey, they must indicate at least one category of activities (a. complete math problems; b. dream; c. express affection; d. swear; e. watch TV; f. read; g. write; h. speak at home) that they prefer in Spanish. (3) Their score on the Spanish vocabulary test was above 52/90. (4) Their score on the Boston naming test was above 24/60. (5) Their accuracy in the lexical decision task was above 75%.

Following these guidelines, none of the 22 participants were removed.

#### 3.1.2. Procedure

Upon arrival, participants were greeted in Spanish and were given the consent form. To assess their experience and proficiency in Spanish, we used three tools: (1) a language history survey, (2) a Spanish vocabulary test that was administered on paper, in which they had to indicate if a string of letters that looked Spanish was indeed a word; and (3) the Boston naming test, which consists of a series of pictures that participants were asked to name in Spanish as quickly and accurately as possible.

The procedure of the masked translation priming experiment was the same as that of Experiment 1 (see Figure 1).

The total time it took to complete the Spanish proficiency/experience and the lexical decision experiment ranged between 30 and 45 min. Upon completion of the entire study, the participants were thanked and given a USD 15 gift card.

#### 3.1.3. Data Analysis

The data inclusion/exclusion plan was as follows: we pre-planned. for 20 participants with the language history described above. As in Experiment 1, the criterion to use the data was 75% correct overall performance, which was achieved by all participants. Given that the trial timed out at 2 s, we decided not to set cutoffs for the RTs.

The data analysis plan was as follows: 1. We first examined if there was evidence for a masked priming effect on the RT, and if so, in which direction. We did this using Bayes Factors as our tool for statistical inference. 2. We drew delta plots and conditional accuracy functions for the latency data. 3. We interpret these results within the evidence accumulation framework.

### 3.2. Results

Table 3 shows the mean RT and the mean accuracy for all types of stimuli.

As can be seen, the high accuracy across all the conditions indicated that participants were able to carry out the task accurately and with RTs in line with other published data. Numerically, the priming effects were evident and the sizes of the priming effects were 21 ms for identity primes, 29 ms for cognates, and 12 ms for non-cognates (all faster compared to unrelated controls).

To compare the data from this experiment to that from Experiment 1, we summarized the average reaction time (RT) for correct responses and the overall accuracy per participant. The data from this experiment were quite similar to that from Experiment 1. The participants in the present experiment were slightly faster overall, with a mean decrease of 16 milliseconds (ms). However, the Bayes factor supports the null model with a value of $BF_{01}=3.11$.

Regarding accuracy, the participants in this study demonstrated slightly higher accuracy compared to Experiment 1, with an increase of 0.037. In this case, the Bayes factor favors the alternative model with a value of $BF_{10}=3.72$.

Like in Experiment 1, we considered three models: (i) a null model ($M_{0}$) that posits the absence of a priming effect, included for the sake of completeness; (ii) a main effects only model ($M_{m}$) that assumes the presence of a priming effect but no interaction with the type of translation prime (cognate vs. non-cognate); and (iii) a full model ($M_{1}$) that includes an interaction term between the type of translation and priming effects.

Based on the computed Bayes Factors (BFs) in this study, we can conclude that the null model, which suggests the absence of a priming effect, should be rejected. The BF for the main effect model compared to the null model was 3, indicating some evidence in favor of the former. However, the comparison between the full model and the main effect model was more relevant to the present research question. The BF for the main effect model relative to the full model was 4021, indicating evidence against an interaction.

### 3.3. Distributional Analyses

**Figure 3:**
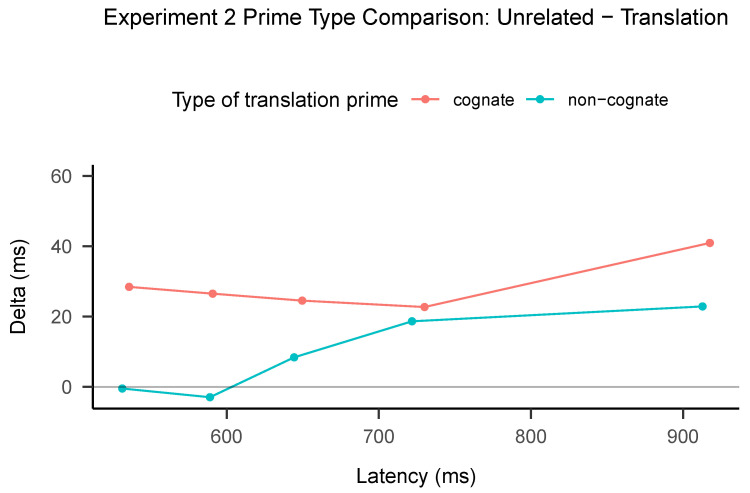
Delta plot for Experiment 2.

As can be seen in Figure 3, the delta plots for both cognate and non-cognate translation primes exhibited differences. The delta plot for cognate primes shows a similar pattern to the other delta plots observed in Experiment 1, characterized by a flat line. In contrast, the non-cognate line displays distinct behavior relative to other masked priming studies, as it increases over time.

In contrast with Experiment 1, only the delta plot for the cognates has the same main feature as the delta plots found by Gomez and Perea [6] and Angele et al. [5]—a flat line. The non-cognates do not show facilitation at the early quantiles; importantly, given the lack of support for the interaction in the Bayes Factors, the difference in the delta plot can be suggestive at best.

## 4. Discussion

This study aimed to investigate the nature of lexical access in bilingual individuals through a masked priming lexical decision task. The research focused on translation priming and explored the effects of cognate and non-cognate translation primes in Spanish and targets in English. The fastest RTs were for identity cognates and the slowest for unrelated primes. This phenomenon has been discussed in previous literature as facilitation [15,16,17,18,19]. We interpret this finding within an evidence accumulation account. The evidence gathered in these two experiments warrants considering the application of the savings hypothesis [3] to translation priming. This explanation has been prominent in the monolingual literature, explaining the encoding advantage that related orthographic and/or semantic primes have on the cognitive processing of targets. We found evidence of savings in bilingual priming, particularly in sequential bilinguals who are more sensitive to the prime as they are more fluent in Spanish reading. Overall, the more similar the target that the participant is responding to with the prime, the greater advantage they have because the lexical entry has already begun [3,5], even breaking language barriers.

Previous research has shown that orthographic representations of cognates, such as “plato” and “PLATE”, are co-activated, leading to facilitated word recognition through the shared semantic representation [13]. However, it is worth noting that inhibition can occur in bilinguals when switching languages, and this phenomenon has been extensively investigated. The inhibitory control model (ICM) suggests that switching tasks are inhibitory regardless of whether a code switch is involved [17]. The cost associated with inhibition is considered external to language-specific mechanisms and is attributed to executive control factors, such as decision-making in laboratory tasks or language production. Language switching inhibition may be related to the activation of the language schema for a specific language, requiring the access of a new schema when switching languages [18]. Top-down inhibition has been observed when bilinguals switch from the inappropriate language to the target language due to the mechanism controlling the relative lexical activation of each language, referred to as “language nodes” [15]. The language node mechanism shares similarities with general cognitive control models, such as those used for attention. However, in the present study, compelling evidence for inhibition in the masked priming process was not found. While clear cross-language inhibitory processes exist in language production, the methodology used here did not reveal evidence of inhibition from translation primes. Further research is needed to explore if there is a baseline inhibition from Spanish to English in all the conditions employed in this study, considering the inherent language shift that occurs.

Although both sequential bilinguals and heritage speakers show translation priming effects, and the shapes of the delta plots are not significantly different, we find both populations to be of interest for future research. This study focused solely on cognitive functions involved when these two populations are primed with a Spanish word followed by a target string of letters consisting of English words or non-words. Importantly, while the contribution of this article is centered on the impact of the priming manipulation on latency distributions, it must be noted that we only explored a relatively small portion of the priming landscape. For instance, future studies could consider using non-masked primes, different language configurations in the prime and target (e.g., Spanish targets and English primes), or manipulating the SOAs of the prime. Each of these manipulations explores distinct theoretical questions, and analyzing distributional features of response times would ideally provide insights into these and related issues.

Overall, the findings of this study offer valuable insights into the nature of lexical access in bilingual individuals. Through an examination of translation priming effects and the application of perceptual accumulation models, the study contributes to our understanding of how languages interact in bilinguals. The results support the savings account hypothesis, which suggests that a related masked prime provides an advantage in processing the target compared to an unrelated prime. This advantage can be seen in the delta plots, indicating an encoding advantage for related primes. While there are various cognitive architectures that can explain this head start, the present work, along with related findings with monolinguals, suggests an encoding advantage (savings). Notably, the study’s finding that the savings account can apply to cross-language priming even with non-cognate primes implies the presence of a small but genuine semantic component in the encoding process that transcends the language barrier. Bilingual selection and control occur at an abstract level and remain encapsulated there, as evidenced by the absence of cascading effects in the decision-making process. Importantly, the assumption of an encapsulated encoding process is a constraint imposed by the use of evidence accumulation models as our explanatory device. Other models might not share this constraint and might interpret the findings differently.

It is important to note that the present study focused on sequential bilinguals and heritage speakers of Spanish, highlighting the variability in bilingual populations and the need for further investigations with diverse bilingual samples.

## Figures and Tables

**Table 1:** Type of primes used in this study.

Type of Priming	Prime	Target
Identity Cognate: Translation prime with an orthographically identical word	doctor	DOCTOR
Non-identity Cognate: Translation primes that are orthographically similar but not identical	sal	SALT
Non-cognate: Translation primes that are not orthographically similar	silla	CHAIR
Unrelated: Non translation	casa	PLATE

**Table 2:** Summary of findings from Experiment 1.

	Translation Prime		Unrelated Control		Difference	
					Unrelated-Translation	
**Type of Translation Prime**	**Mean RT**	**Mean Accurarcy**	**Mean RT**	**Mean Accurarcy**	**RT**	**Accuracy**
Identity Cognate	643	0.941	714	0.958	61	0.017
Non-identity Cognate	695	0.929	730	0.907	35	−0.022
Non-cognate	732	0.867	741	0.861	9	−0.006

**Table 3:** Summary of findings from Experiment 2.

	Translation Prime		Unrelated Control		Difference	
					Unrelated-Translation	
**Type of Translation Prime**	**Mean RT**	**Mean Accurarcy**	**Mean RT**	**Mean Accurarcy**	**RT**	**Accuracy**
Identity Cognate	675	0.980	696	0.957	21	−0.023
Non-identity Cognate	687	0.944	716	0.924	29	−0.020
Non-cognate	687	0.940	699	0.943	12	0.003

## Data Availability

Data, scripts and materials are available at OSF: https://osf.io/str9c/.

## Abbreviations

The following abbreviations are used in this manuscript:
  RT	Response Time
  DDM	Drift-Diffusion Model
  SOA	Stimulus Onset Asynchrony

## References

[1] Balota D.A., Chumbley J.I. (1984). Are lexical decisions a good measure of lexical access? The role of word frequency in the neglected decision stage. J. Exp. Psychol. Hum. Percept. Perform..

[2] Forster K.I., Davis C. (1984). Repetition priming and frequency attenuation in lexical access. J. Exp. Psychol. Learn. Mem. Cogn..

[3] Forster K.I. (1998). The pros and cons of masked priming. J. Psycholinguist. Res..

[4] Grainger J. (2008). Cracking the orthographic code: An introduction. Lang. Cogn. Process..

[5] Angele B., Baciero A., Gómez P., Perea M. (2023). Does online masked priming pass the test? The effects of prime exposure duration on masked identity priming. Behav. Res. Methods.

[6] Gomez P., Perea M. (2020). Masked identity priming reflects an encoding advantage in developing readers. J. Exp. Child Psychol..

[7] Lee Y., Jang E., Choi W. (2018). L2-L1 translation priming effects in a lexical decision task: Evidence from low proficient Korean-English bilinguals. Front. Psychol..

[8] Chaouch-Orozco A., Alonso J.G., Rothman J. (2021). Individual differences in bilingual word recognition: The role of experiential factors and word frequency in cross-language lexical priming. Appl. Psycholinguist..

[9] de Groot A.M., Nas G.L. (1991). Lexical representation of cognates and noncognates in compound bilinguals. J. Mem. Lang..

[10] Jiang N. (1999). Testing processing explanations for the asymmetry in masked cross-language priming. Biling. Lang. Cogn..

[11] Xia V., Andrews S. (2015). Masked translation priming asymmetry in Chinese-English bilinguals: Making sense of the Sense Model. Q. J. Exp. Psychol..

[12] Duñabeitia J.A., Perea M., Carreiras M. (2010). Masked Translation Priming Effects with Highly Proficient Simultaneous Bilinguals. Exp. Psychol..

[13] Dijkstra T., Wahl A., Buytenhuijs F., Van Halem N., Al-Jibouri Z., De Korte M., Rekké S. (2019). Multilink: A computational model for bilingual word recognition and word translation. Biling. Lang. Cogn..

[14] Gomez P., Perea M., Ratcliff R. (2013). A diffusion model account of masked versus unmasked priming: Are they qualitatively different?. J. Exp. Psychol. Hum. Percept. Perform..

[15] Chauncey K., Grainger J., Holcomb P.J. (2008). Code-switching effects in bilingual word recognition: A masked priming study with event-related potentials. Brain Lang..

[16] Litcofsky K.A., Van Hell J.G. (2017). Switching direction affects switching costs: Behavioral, ERP and time-frequency analyses of intra-sentential codeswitching. Neuropsychologia.

[17] Ong G., McKague M., Weekes B., Sewell D.K. (2019). Diffusing the bilingual lexicon: Task-based and lexical components of language switch costs. Cogn. Psychol..

[18] Bobb S.C., Wodniecka Z. (2013). Language switching in picture naming: What asymmetric switch costs (do not) tell us about inhibition in bilingual speech planning. J. Cogn. Psychol..

[19] Dijkstra T., Van Heuven W.J. (2002). The architecture of the bilingual word recognition system: From identification to decision. Biling. Lang. Cogn..

[20] Ratcliff R. (1978). A theory of memory retrieval. Psychol. Rev..

[21] Ratcliff R., Gomez P., McKoon G. (2004). A diffusion model account of the lexical decision task. Psychol. Rev..

[22] Gomez P., Ratcliff R., Perea M. (2007). A model of the go/no-go task. J. Exp. Psychol. Gen..

[23] Gomez P., Perea M. (2014). Decomposing encoding and decisional components in visual-word recognition: A diffusion model analysis. Q. J. Exp. Psychol..

[24] Yang H., Jared D., Perea M., Lupker S.J. (2021). Is letter position coding when reading in L2 affected by the nature of position coding used when bilinguals read in their L1?. Mem. Cogn..

[25] De Wit B., Kinoshita S. (2015). The masked semantic priming effect is task dependent: Reconsidering the automatic spreading activation process. J. Exp. Psychol. Learn. Mem. Cogn..

[26] deBruin A. (2019). Not All Bilinguals Are the Same: A Call for More Detailed Assessments and Descriptions of Bilingual Experiences. Behav. Sci..

[27] Costa A., Sebastián-Gallés N. (2014). How does the bilingual experience sculpt the brain?. Nat. Rev. Neurosci..

[28] Valdés G. (2005). Bilingualism, heritage language learners, and SLA research: Opportunities lost or seized?. Mod. Lang. J..

[29] Brysbaert M., Stevens M. (2018). Power Analysis and Effect Size in Mixed Effects Models: A Tutorial. J. Cogn..

[30] Balota D.A., Yap M.J., Hutchison K.A., Cortese M.J., Kessler B., Loftis B., Neely J.H., Nelson D.L., Simpson G.B., Treiman R. (2007). The English lexicon project. Behav. Res. Methods.

[31] Lund K., Burgess C. (1996). Producing high-dimensional semantic spaces from lexical co-occurrence. Behav. Res. Methods Instrum. Comput..

[32] Yarkoni T., Balota D., Yap M. (2008). Moving beyond Coltheart’s N: A new measure of orthographic similarity. Psychon. Bull. Rev..

[33] Morey R.D., Rouder J.N. (2011). Bayes factor approaches for testing interval null hypotheses. Psychol. Methods.

[34] Ridderinkhof K.R., van den Wildenberg W.P.M., Wijnen J., Burle B., Posner M.I. (2004). Response inhibition in conflict tasks is revealed in delta plots. Cognitive Neuroscience of Attention.

[35] De Jong R., Liang C.C., Lauber E. (1994). Conditional and unconditional automaticity: A dual-process model of effects of spatial stimulus-response correspondence. J. Exp. Psychol. Hum. Percept. Perform..

[36] Ratcliff R. (1979). Group reaction time distributions and an analysis of distribution statistics. Psychol. Bull..

